# HIF-1α and HIF-2α induced angiogenesis in gastrointestinal vascular malformation and reversed by thalidomide

**DOI:** 10.1038/srep27280

**Published:** 2016-06-01

**Authors:** Nan Feng, Haiying Chen, Sengwang Fu, Zhaolian Bian, Xiaolu Lin, Li Yang, Yunjie Gao, Jingyuan Fang, Zhizheng Ge

**Affiliations:** 1 Key Laboratory of Gastroenterology & Hepatology, Ministry of Health, Division of Gastroenterology & Hepatology, Ren Ji Hospital, School of Medicine, Shanghai Jiao Tong University, Shanghai Institute of Digestive Diseases, 145 Middle Shandong Road, Shanghai 200001, China; 2 Department of gastroenterology and hepatology, Nantong Institute of liver diease, Nantong Third people’s Hospital, Nantong 223006, China

## Abstract

Thalidomide is used in clinical practice to treat gastrointestinal vascular malformation (GIVM), but the pathogenesis of GIVM is not clear. Hypoxia inducible factor 1 alpha (HIF-1α) and 2 alpha (HIF-2α/EPAS1) are in the same family and act as master regulators of the adaptive response to hypoxia. HIF-1α and HIF-2α are up-regulated in vascular malformations in intestinal tissues from GIVM patients, but not in adjacent normal vessels. Therefore, we investigated the role of HIF-1α and HIF-2α during angiogenesis and the mechanism of thalidomide action. *In vitro* experiments confirmed that vascular endothelial growth factor (VEGF) was a direct target of HIF-2α and that HIF-1α and HIF-2α regulated NOTCH1, Ang2, and DLL4, which enhanced vessel-forming of endothelial cells. Thalidomide down-regulated the expression of HIF-1α and HIF-2α and inhibited angiogenesis. *In vivo* zebrafish experiments suggested that HIF-2α overexpression was associated with abnormal subintestinal vascular (SIV) sprouting, which was reversed by thalidomide. This result indicated that thalidomide regulated angiogenesis via the inhibition of HIF-1α and HIF-2α expression, which further regulated downstream factors, including VEGF, NOTCH1, DLL4, and Ang2. The abnormally high expression of HIF-1α and HIF-2α may contribute to GIVM.

Gastrointestinal vascular malformation (GIVM) is a common vascular lesion of the gastrointestinal tract that often underlies unexplained gastrointestinal bleeding, especially in the elderly^1^. Age is the only identified epidemiological factor for GIVM, which often leads to acute bleeding and chronic anaemia and significantly influences the quality of life. Many patients suffer from multiple lesions, and traditional treatments, such as angiographic embolisation and surgical excision, often result in poor outcomes.

Previous studies suggested that GIVM was associated with abnormal angiogenesis^2^. Angiogenesis primarily involves three signalling pathways: the VEGF-VEGF receptor pathway, the angiopoietin (Ang)-Tie2 axis, and the Notch pathway^3^,^4^. The VEGF pathway plays a key role in each step of angiogenesis. Approximately 80% of GIVM occurs in the cecum, but the lower small intestine may also be involved^5^. The cecum exhibits the highest intestinal pressure, and high pressure may lead to hypoxia. One previous study suggested that hypoxia was significantly associated with the development of GIVM^6^.

Hypoxia-inducible factor-1α (HIF-1α) enhances VEGF expression^7^. HIF-1α and HIF-2α belong to the same family. These proteins are the master regulators of oxygen homeostasis and play a vital role in the pathogenesis of different hypoxia-related diseases. HIF-1α controls more than 100 genes, and more than 2% of all human genes in endothelial cells may be directly or indirectly regulated by HIF-1α^8^. Selective HIF-2α-responsive genes are also very important in the regulation of hypoxia^9^, and previous studies demonstrated differences between HIF-1α and HIF-2α. These proteins are involved in normal development and pathological conditions, such as tumours and vascular diseases. One of our previous studies found that HIF-1α, Ang-2, DLL4, and Notch1 participated in the development of GIVM^10^. Another previous work suggested that the angiogenesis inhibitor thalidomide effectively treated GIVM-associated gastrointestinal bleeding^11^,^12^.

Therefore, the present study investigated the pathogenesis of GIVM and the mechanisms of thalidomide treatment of GIVM, especially the differences and relationship of HIF-1α and HIF-2α in the angiogenesis of GIVM specimens, human umbilical vein endothelial cells (HUVECs), and the role of HIF-2α in zebrafish.

## Results

### HIF-2α expression was up-regulated in vascular malformation lesions compared to normal intestinal vasculature

Intestinal specimens from 8 patients who were suffering from acute gastrointestinal bleeding and underwent bowel resection at the Renji Hospital, Shanghai Jiaotong University between November 2004 and March 2011 were selected. Specimens were taken from four males and four females aged 42–72 years (median, 62 years). The median number of bleeding events was 6 per year (range, 1–9 events per year). Their median haemoglobin level was 72 g/L (range, 45–106 g/L). The median volume of blood transfusion was 1800 ml (range, 400–4800 ml). Four of the 8 patients had lesions in the small intestine, and four patients had lesions in the right colon. Six patients had lesions at a single site, and two patients had lesions at multiple sites. The Ethics Committee of the Renji Hospital, Shanghai Jiaotong University approved this study. The committee waived the need for individual consent because of the retrospective nature of the study.

**Figure 1:**
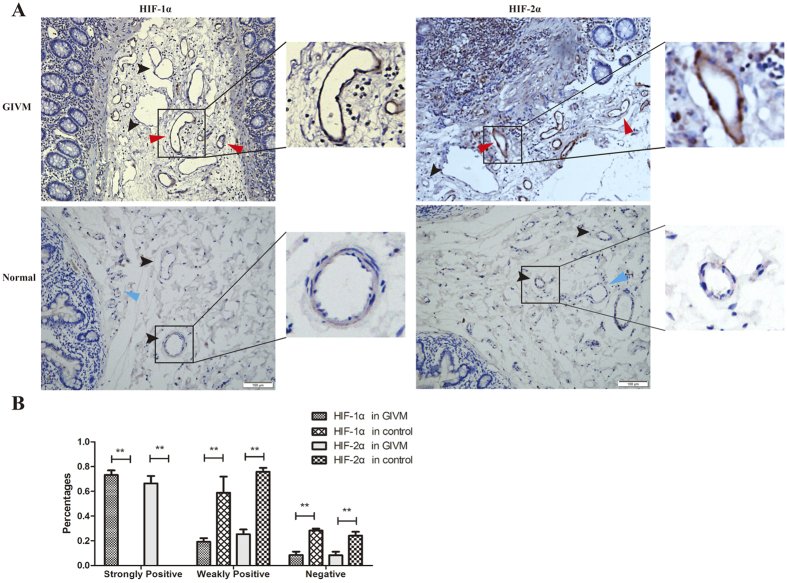
(**A**) The expression of HIF-1α and HIF-2α in gastrointestinal vascular malformations and normal vessels. Red arrow: strongly positive; Black arrow: weakly positive; Blue arrow: negative. (**B**) Percentages of positive and negative vessels in GIVM and normal tissues. **P < 0.01.

GIVM lesions presented with tortuous and dilated arterioles, venules, and capillaries. Immunohistological examination revealed that HIF-1α and HIF-2α exhibited high immunoreactivity in the cytoplasm and nucleus in GIVM specimens, but the immunogenicity of vessels in normal tissue was low or negative (Fig. 1A). Normal and abnormal vessels were not recognised by structure alone. Therefore, we defined strongly positive as a positive area that included more than 50% of the endothelial cell, weakly positive as a positive area that included less than 50% of the cell, and negative as no positive area. The calculation was performed in 5 random non-overlapping images for each group under a 200× microscopic field. The results revealed that 73.2 ± 8.4% and 66.4 ± 13.5% of vessels in GIVM tissues were strongly positive for HIF-1α and HIF-2α, respectively. Weakly positive HIF-1α and HIF-2α vessels were major findings in normal tissues (Fig. 1B).

### Enhanced angiogenesis under hypoxia

**Figure 2:**
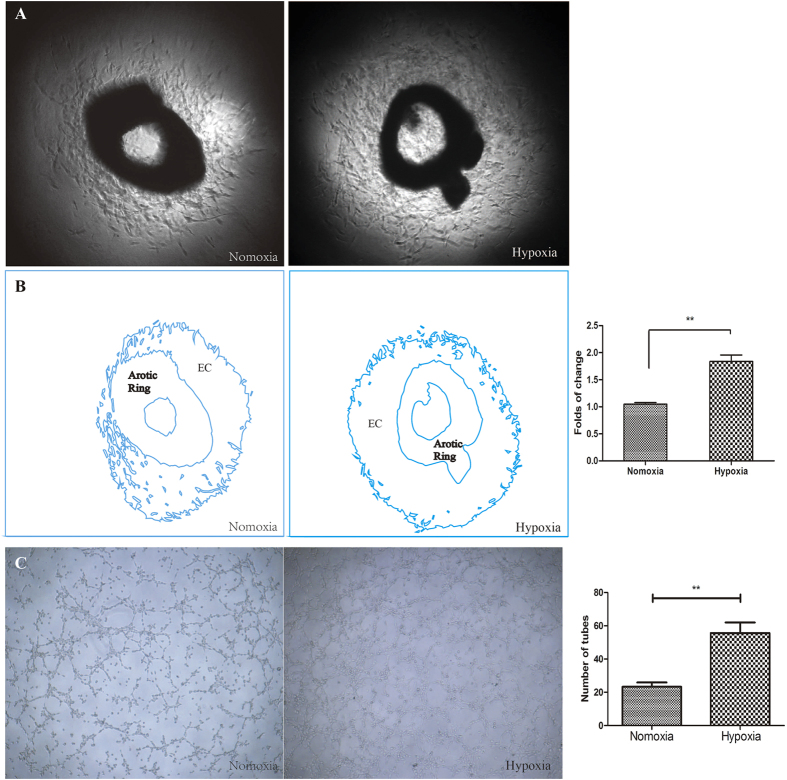
The effect of hypoxia on angiogenesis was assessed using mouse thoracic aortas and HUVECs. (**A**) Aortic ring assays revealed increased EC proliferation under hypoxia. (**B**) The outline in (**A**) was drawn using Adobe Illustrator software, and the area was calculated. (**C**) Representative images of tube formation under normoxia and hypoxia. Tube formation was enhanced under hypoxia for 6 h. (EC: Endothelial Cells) **P < 0.01 *vs.* normoxia.

Thoracic aortas of mice were collected to observe variations in angiogenesis, and these tissues exhibited enhanced angiogenesis of 1.84 ± 0.12-fold (P < 0.01 vs. normoxia) under hypoxic conditions. Tube formation was enhanced under hypoxic conditions compared to normoxia at 6 h (P < 0.01) (Fig. 2).

### Hypoxia up-regulated HIF-1α and HIF-2α and promoted angiogenesis processes in HUVECs

HUVEC was widely used to study vascular malformation (e.g. cerebral cavernous malformation)^13^,^14^ and cancer angiogenesis^15^,^16^,^17^. HUVECs purchased from the Institute of Cell Biology of the Chinese Academy of Sciences (Shanghai, China) were also used in our previous *in vitro* studies^12^,^18^.

**Figure 3:**
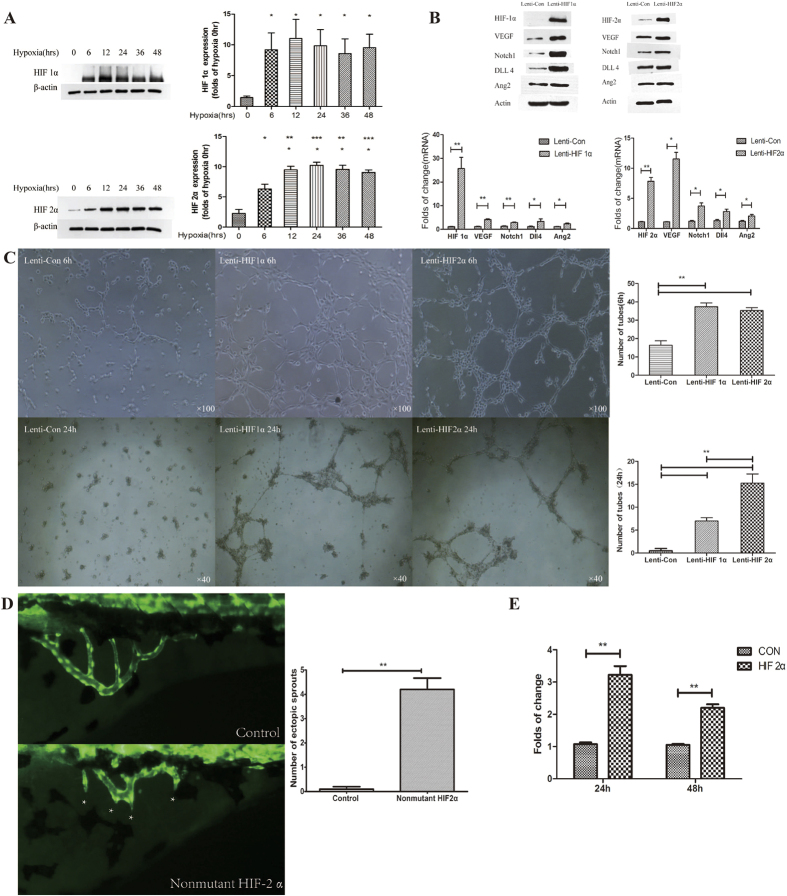
(**A**) Western blot determinations of HIF-1α and HIF-2α expression at different time points of hypoxia. *P < 0.05, **P < 0.01 *vs.* 0 hour. (**B**) The effect of HIF-1α and HIF-2α overexpression on the expression of VEGF, Notch1, DLL4, and Ang2. Western blot and RT-PCR demonstrated that HIF-1α and HIF-2α overexpression increased the expression of VEGF, Notch1, DLL4, and Ang2 protein and mRNA. *P < 0.05, **P < 0.01 *vs.* control. (**C**) Influence of HIF-1α and HIF-2α overexpression on angiogenesis 6 and 24 h after transfection of Lenti-HIF-1α and Lenti-HIF2α. Tube formation was enhanced 6 and 24 h after transfection. **P < 0.01 *vs.* control. (**D**) Fluorescence microscope observations of subintestinal vein sprouting in normal and HIF-2α-overexpressing zebrafish. *Indicates subintestinal vascular sprouts. HIF-2α overexpression significantly increased the number of subintestinal vascular sprouts. **P < 0.01 *vs.* control plasmid. (**E**) Dual luciferase reporter gene assay demonstrated that HIF-2α enhanced VEGF promoter activity. **P < 0.01 *vs.* control plasmid.

Western blot investigated HIF-1α and HIF-2α expression in HUVECs after different hypoxia exposure times. HIF-1α and HIF-2α expression initially increased with the time of hypoxia and peaked at 12 h and 24 h, respectively (P < 0.001 vs. 0 hour). HIF-1α decreased gradually thereafter, and HIF-2α remained stable at a relatively high level (Fig. 3A).

Lenti-HIF1α and Lenti-HIF2α lentivirus vectors were constructed and transfected into HUVECs to examine the influence of HIF1α and HIF-2α on angiogenesis. Tube formation processes were enhanced at 6 h, but these processes ceased at 24 h, and the formed tubes disappeared in the control group. However, the number of tubes was abundant in HIF1α or HIF-2α overexpressing cells at 6 h, and the process remained active at 24 h (P < 0.01 vs. control lentivirus) (Fig. 3B).

These results demonstrated that hypoxia induced the up-regulation of HIF-1α and HIF-2α and that the latter promoted angiogenesis processes in HUVECs. Further studies on the downstream effects of HIF-1α and HIF-2α were performed. VEGF, Notch1, DLL4, and Ang2 were detected using Western blotting and RT-PCR in HIF1α and HIF-2α overexpressing HUVEC strain. HIF-1α and HIF-2α overexpression increased the expression of VEGF, Notch1, DLL4, and Ang2 (P < 0.05 vs. control plasmid) (Fig. 3C).

### HIF-2α induced subintestinal vascular sprouting in a zebrafish model

The role of HIF-1α in angiogenesis was investigated thoroughly, but the role of HIF-2α in this process was not known. The dual-luciferase reporter gene assay demonstrated that HIF-2α increased the activity of the VEGF promoter (Fig. 3E), which suggests that VEGF was a direct target of HIF-2α.

Zebrafish vascular model was described by Serbedzija *et al*. in 1999^19^, and it has became one of the main whole animal model for angiogenesis study^20^,^21^,^22^. Therefore, a zebrafish vascular model was selected to evaluate the influence of HIF-2α overexpression on vascular development *in vivo*, which cannot be observed in human intestinal tissue. HIF-2α-overexpressing lentiviruses were injected into zebrafish embryos, and vascular development was observed using a fluorescence microscope. HIF-2α overexpression induced subintestinal vascular sprouting in the zebrafish model (P < 0.0001 vs. control plasmid) (Fig. 3D).

### Thalidomide inhibited HIF-1α and HIF-2α *in vitro*

**Figure 4:**
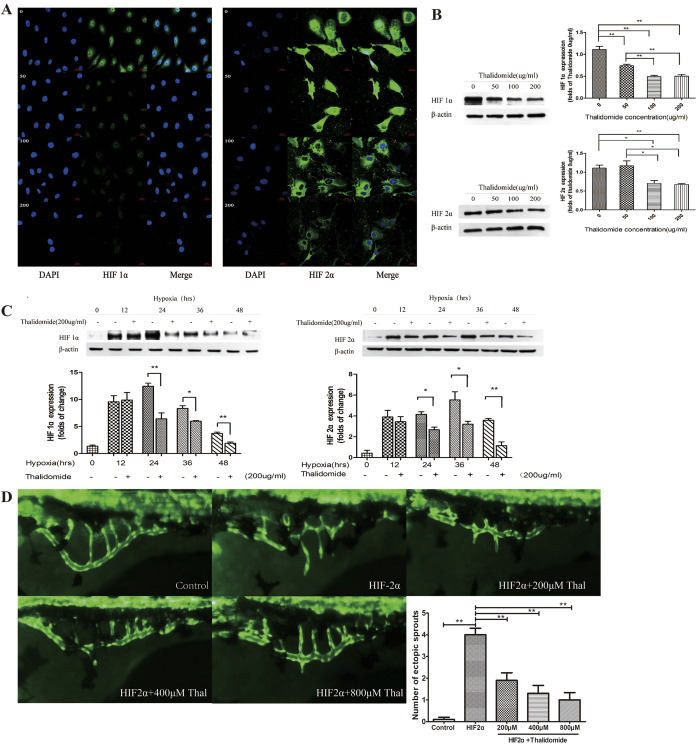
The inhibitory effect of thalidomide. (**A**) Immunofluorescence indicated that HIF-1α and HIF-2α expression was down-regulated by thalidomide at different concentrations. (**B**) Western blots demonstrated that the expression of HIF-1α and HIF-2α decreased with 100 and 200 μg/ml of thalidomide. *P < 0.05, **P < 0.01. (**C**) Western blots demonstrated that thalidomide at 200 μg/ml inhibited the expression of HIF-1α and HIF-2α in HUVECs after hypoxic treatment for 24, 36, and 48 h. *P < 0.05, **P < 0.01. (**D**) Fluorescence microscope observations of the effect of thalidomide at different concentrations on vascular development in zebrafish with HIF-2α overexpression. **P < 0.01 *vs.* HIF-2α.

HUVECs cultured under hypoxic conditions were treated with different concentrations of thalidomide (0, 50, 100, and 200 μg/ml). HIF-1α and HIF-2α expression was observed using laser scanning confocal microscopy and Western blotting. The expression of both proteins decreased gradually with increasing thalidomide concentrations (Fig. 4A,B).

The effect of 200 μg/ml of thalidomide on HIF-1α and HIF-2α expression was investigated at different hypoxia condition times (0, 12, 24, 36, and 48 h). Thalidomide at 200 μg/ml effectively inhibited HIF-2α expression after 24 h of hypoxia (P < 0.05) (Fig. 4C).

### Thalidomide reversed the angiogenesis process via inhibition of HIF-2α *in vivo*

Zebrafish embryos overexpressing HIF-2α were treated with different thalidomide concentrations (0, 200, 400, and 800 μM). All thalidomide concentrations decreased the number of subintestinal vascular sprouts (P < 0.0001 vs. 0 μM of thalidomide). The vascular phenotype of zebrafish treated with 800 μM of thalidomide was very similar to normal zebrafish (Fig. 4D).

**Figure 5:**
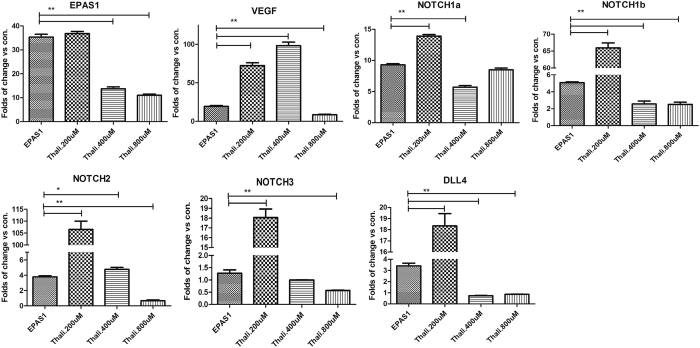
The expression of HIF-2α (EPAS1), VEGF, Notch1a, Notch1b, Notch2, Notch3, and DLL4 in the zebrafish model. *P < 0.05, **P < 0.01.

RT-PCR analysis demonstrated that HIF-2α overexpression up-regulated VEGF, NOTCH1a, NOTCH1b, NOTCH2, NOTCH3, and DLL4 (P < 0.05 vs. control). Thalidomide (200 μM and 400 μM) inhibited the expression of all factors except VEGF, but 800 μM of thalidomide strongly inhibited VEGF (Fig. 5). These results indicated that thalidomide reversed the effect of HIF-2α overexpression *in vivo*.

## Discussion

This study examined the pathogenesis of GIVM and the mechanisms of thalidomide treatment for GIVM, especially the possible involvement of HIF in the angiogenesis of GIVM specimens, human umbilical vein endothelial cells (HUVECs), and zebrafish.

One previous study suggested a significant association of hypoxia with GIVM^6^, and another study demonstrated high HIF-1α expression in GIVM patients^10^. Various hypoxic conditions stimulate angiogenesis, which was also demonstrated in our study. Mouse thoracic aortas under hypoxic conditions *in vitro* exhibited enhanced angiogenesis in tube formation experiments. These results suggest that one of the reasons for GIVM onset is hypoxia-induced HIF up-regulation. HIF-1α and HIF-2α were highly expressed in GIVM specimens compared to self-normal vascular control specimens. However, several differences between HIF-1α and HIF-2α were observed. HIF-2α expression in HUVECs increased gradually with increasing hypoxia exposure, peaked at 12–24 h, and remained at a relatively high level despite a slight decrease. HIF-1α was activated earlier than HIF-2α, peaked at 12 h, and decreased gradually, which indicated that HIF-1α was a “rapid, relatively short-term” factor adaptation to hypoxia. In contrast, HIF-2α was a “slow, relatively long-term” factor.

High HIF-1α and HIF-2α expression induces the expression of many angiogenesis-regulating genes^23^,^24^,^25^. Our study confirmed that HIF-1α and HIF-2α overexpression significantly enhanced the protein and mRNA expression of VEGF, Notch1, DLL4, and Ang2, which are key factors during angiogenesis. Notably, we found that HIF-2α activated downstream factors much stronger than HIF-1α, especially VEGF, up to 11.52 ± 1.94-fold of the control group. HIF-1α induced a 4.06 ± 0.68-fold greater activation. The dual luciferase reporter gene assay also found that HIF-2α increased activity of the VEGF promoter. These results suggest a role for HIF-2α in the pathological processes of GIVM, not only HIF-1α. The effect of HIF-2α was more persistent.

Therefore, we constructed an animal model to investigate our hypothesis. The role of HIF-2α in angiogenesis was confirmed in several animal models^26^,^27^. The present study used a zebrafish model because of its high fecundity, transparent embryos, *in vitro* development, and rapid growth. The most important advantage of zebrafish was the availability of dynamic observation. The blood vessels of zebrafish can also be specifically labelled using green fluorescent protein to allow direct observation of blood vessel changes. Zebrafish embryos were microinjected with a plasmid vector that overexpressed HIF-2α, and vascular sprouting was observed under a fluorescence microscope. The results revealed that HIF-2α overexpression significantly increased the number of vascular sprouts. The success of animal modelling supported our hypothesis.

Historically, thalidomide was used to treat pregnant women with nausea or vomiting, but this use was prohibited because thalidomide was associated with congenital limb defects in newborns^28^. Recent studies reported that thalidomide may be used to treat myeloma and Crohn’s disease^29^ because it inhibited inflammation and regulated immune reactions. Thalidomide is an inhibitor of angiogenesis, and it reduced the expression of basic fibroblast growth factor (FGF) in rabbit cornea and decreased serum levels of VEGF in patients with severe intestinal haemorrhage^30^. Our previous clinical study indicated that thalidomide was effective for GIVM-associated bleeding^11^. The present results suggest that thalidomide efficacy may be attributed to the regulation of abnormal HIF-1α and HIF-2α expression. Different thalidomide concentrations inhibited HIF-1α and HIF-2α expression under hypoxia *in vitro*. The maximum thalidomide concentration was 200 μg/ml when dissolved in DMSO, and this concentration was used for the subsequent experiments on HIF-2α expression in hypoxic cells. The results demonstrated that 200 μg/ml thalidomide inhibited HIF-1α and HIF-2α expression after 24, 36, and 48 hours of hypoxia. Different thalidomide concentrations inhibited vascular sprouting in the intestine of zebrafish. Notably, zebrafish treated with 800 μM thalidomide exhibited a vascular phenotype that was very similar to healthy controls. Detection of downstream factors, such as VEGF, NOTCH, DLL4, and Ang2, demonstrated that thalidomide influenced the expression of these factors *in vitro* and in zebrafish. These results indicated that thalidomide regulated angiogenesis via inhibition of HIF-2α.

Hypoxia up-regulates HIF-1α and HIF-2α, and HIF-2α promotes downstream factors, such as VEGF. Thalidomide reversed the vascular malformations. However, we know less regarding the conditions that produce the anoxic intestinal environment. Local pressure changes are one possible explanation, but this hypothesis requires further study.

In conclusion, abnormal HIF-1α and HIF-2α expression is involved in GIVM. The influence of HIF-1α on angiogenesis was investigated *in vitro*, and HIF-2α was evaluated *in vitro* and *in vivo* (zebrafish model). Thalidomide influenced angiogenesis by inhibiting HIF-1α and HIF-2α expression, which affected downstream factors, including VEGF, NOTCH, DLL4, and Ang2.

## Materials and Methods

### Immunohistochemistry

Samples were fixed in 10% formalin, embedded in paraffin, and sectioned to a thickness of 4 μm. Sections were dewaxed in xylene, hydrated in alcohol, incubated with 3% H_2_O_2_ to inactivate endogenous peroxidase, and incubated with a citrate solution for antigen retrieval. Sections were blocked with goat serum for 30 min and incubated with a mouse-anti-HIF-2α antibody (1:200, Abcam, Cambridge, UK) at 4 °C overnight. Sections were incubated with a secondary antibody (Shanghai Long Island Biotec. Co., Ltd., Shanghai, China) and DAB (Fuzhou Maixin Biotechnology Development Co., Ltd., Fuzhou, Fujian Province, China), followed by haematoxylin staining and resin mounting. HIF-2α expression was observed under a light microscope. Vascular endothelial cells showing a yellow- or brown-coloured cytoplasm were deemed positive.

### Angiogenesis of mouse thoracic aortas

Matrigel was thawed at 4 α prior to experiments. Mice aged 8 weeks (purchased from the Institute of Model Animals, Nanjing University) were euthanized using cervical dislocation, and their thoracic aortas were isolated from surrounding fat tissues and branch vessels. Residual blood was removed, and the aorta was sliced into rings with a width of 0.5–1 mm. The rings were immersed in ice-cold PBS.

Matrigel (50 μL; BD Biosciences, Franklin Lakes, NJ, USA) was added to 10 wells of two 96-well plates and incubated at 37 °C for 15 min to allow solidification. Aortic rings were placed horizontally on the gel with one ring per well. Another 50 μL of Matrigel was added and incubated at 37 °C for 15 min for solidification. Each well received 200 μL of Opti-MEM medium containing 2.5% FBS and 1% penicillin/streptomycin. One plate was placed in a normoxic incubator (37 °C, 21% O_2_, 5% CO_2_), and the other was placed in a hypoxemic incubator (37 °C, 1% O_2_, 5% CO_2_). The plates were photographed under an inverted microscope when angiogenesis peaked (6–10 d), and ImageJ software (National Institutes of Health, Bethesda, MD, USA) was used for data analysis.

### *In vitro* HUVEC cultures

HUVECs were purchased from the Institute of Cell Biology of the Chinese Academy of Sciences (Shanghai, China). The cells were cultured in Dulbecco’s modified Eagle’s medium (DMEM) (GIBCO, Invitrogen Inc., Carlsbad, CA, USA) with 15% foetal bovine serum (Zhejiang Tianhang Biological Technology Co., Ltd., Hangzhou, Zhejiang Province, China) and 100 U/ml penicillin-streptomycin (Invitrogen, Carlsbad, CA, USA). The cells were passaged every three days and recovered from frozen stock every two months. Cells were seeded at an initial density of 1.0 × 10^5 ^cells/well in a 6-well plate (Corning, Corning, NY, USA). Cells were initially placed in a normoxic incubator for 24 h. Individual plates of cells were incubated in either hypoxic (at 37 °C with 5% CO_2_, 2% O_2_, and 93% N_2_; Changsha Huaxi Electronic Technology, Changsha, Hunan Province, China) or normoxic conditions (at 37 °C with 5% CO_2_ and 95% O_2_; Shel Lab Model 2300, Sheldon Manufacturing, Cornelius, OR, USA)^12^. The expression of HIF-2α in HUVECs was detected using Western blotting at 0, 6, 12, 24, and 48 h of culture under hypoxia.

### Thalidomide treatment of HUVECs

Thalidomide was dissolved in dimethyl sulfoxide (DMSO) (both from Sigma-Aldrich, St. Louis, MO, USA) at 100 and 200 mg/ml and was stored at −4 °C for no more than 4 weeks. Thalidomide was diluted to different concentrations (25, 50, 100, and 200 μg/ml) immediately prior to use. The DMSO concentration was less than 0.1%.

HUVECs underwent a 24-hour synchronization in 6-well plates. HUVECs grown under hypoxia were divided into four groups: 1) the control group (DMSO < 0.1%); 2) the 50 μg/ml thalidomide group; 3) the 100 μg/ml thalidomide group; and 4) the 200 μg/ml thalidomide group. The cells were treated for 24 hours. The expression of HIF-2α in HUVECs was assessed using immunofluorescence and Western blotting. HUVECs were treated with 200 μg/ml of thalidomide and harvested after 0, 12, 24, 36, and 48 h of culture under hypoxia. The expression of HIF-2α in HUVECs was assessed using Western blots.

### Immunofluorescence

HUVECs treated with thalidomide (50, 100, and 200 μg/ml) and hypoxia were harvested, fixed with 4% paraformaldehyde for 10 min, cleared with 0.2% Triton X-100 for 10 min, blocked with BSA for 30 min, and incubated with an anti-HIF-2α antibody (1:200; Abcam, Cambridge, UK) at 4 °C overnight. An anti-rabbit fluorescent antibody was added and incubated in the dark for 30 min. DAPI staining was performed followed by section mounting.

### HIF-2α-overexpressing lentivirus vector

HUVECs at passage 3 were transfected with HIF-1α- and HIF-2α-overexpressing lentivirus or empty vector, and the expression of HIF-1α, HIF-2α, VEGF, NOTCH1, DLL4, and Ang2 were measured using Western blotting and real-time PCR.

### Western blots

HUVECs transfected with HIF-2α-overexpressing lentivirus were collected after different hypoxia treatment times. Cells were lysed (60 μL RIPA lysis solution, RIPA:PMSF = 50:1) on ice for 30 min. The lysate was centrifuged at 12,000 rpm and 4 °C for 5 min. Protein was quantified using a BSA assay, and protein samples (50 μg) were loaded and electrophoresed on a 10% SDS-PAGE gel. Proteins were transferred to a nitrocellulose membrane (Millipore, Bedford, MA, USA), which was blocked using 5% milk for 2 h, and incubated with rabbit-anti-HIF-2α, VEGF, NOTCH1, DLL4, Ang2, and β-actin antibodies (all rabbit polyclonal antibodies from KangChen Bio-tech, Shanghai, China) at 4 °C overnight. An HRP-labelled secondary antibody (1:5000; Cell Signaling Technology, Danvers, MA, USA) was added and incubated at room temperature for 1 h. Chemiluminescence, development, and fixation were performed. ImageJ software (National Institutes of Health, Bethesda, MD, USA) was used to calculate and analyse the OD value of each specific band.

### Real-time PCR

Total RNA was extracted from HUVECs and zebrafish using Trizol (Invitrogen Inc., Carlsbad, CA, USA). The purity and concentration of RNA was measured. RNA was reverse transcribed into cDNA according to the instructions provided with the reverse transcription kit (Takara Bio Inc., Shiga, Japan). RT-PCR (Applied Biosystems, Foster City, CA, USA) was performed to detect the mRNA expression of HIF-1α, HIF-2α, VEGF, Notch1, DLL4, and Ang2 in HUVECs and HIF-2α, VEGF, Notch1a, Notch1b, Notch2, Notch3, and DLL4 in zebrafish. Supplementary material 1 shows the primers for RT-PCR.

### Dual-luciferase reporter gene assay

The promoter sequence of VEGFA (NM_001025366) was obtained from the NCBI web site (National Center of Biotechnology Information) and cloned into the pEZX-PG04 Gaussia luciferase-SEAP dual reporter vector (MPRM11909-PG04, Genecopoeia, Maryland, USA). DH5α-competent cells (100 μL) were added with 5 μL of DNA for plasmid transformation. The transformed cells were spread onto an LB plate containing kanamycin and cultured at 37 °C for 16 h. A total of 20–30 colonies were selected and sequenced using universal primers to screen for positive clones. Positive single clones were inoculated in kanamycin-containing LB liquid medium and cultured for plasmid extraction. Extracted plasmids were transfected into HUVECs and passaged for more than 3 generations. The activity of GLU and SEAP were measured to calculate the standardised signal using the formula: Standardised Gluc activity = relative fluorescence intensity of Gluc/relative florescence intensity of SEAP.

### Angiogenesis determination using the capillary-like tube formation assay

Matrigel (BD Biosciences, Franklin Lakes, NJ, USA) was thawed at 4 °C on the day before experiment. Matrigel and culture medium were mixed (V/V 1:1) in a pre-cooled EP tube and added to a 48-well plate at a volume of 100 μL. The plate was placed in an incubator for 30 min to allow solidification. HUVECs (recovered and cultured to passage 3) transfected with HIF-1α and HIF-2α lentivirus or empty vector were adjusted to a concentration of 6 × 10^4 ^cells/ml and added to corresponding wells at a volume of 100 μL. The 48-well plate was cultured at 37 °C with 5% CO_2_ and 21% O_2_, and tube formation was observed under a microscope 6 and 24 h after seeding.

### Zebrafish

Zebrafish (*Danio rerio*) embryos were obtained from the natural spawning of a wild-type EK line (Ekkwill Farms, Gibonston, FL, USA) that has been inbred for several generations. Zebrafish were raised and maintained under standard conditions^31^. Embryos were staged according to previous studies^32^. The establishment and characterisation of the fli1a-EGFP transgenic lines have been described in a previous study^33^. The Shanghai Research Center For Model Organisms Animal Care and Use Committee approved the animal procedures described below. The procedures were conducted in accordance with the guidelines of the American Veterinary Medical Association’s (AVMA) Panel on Euthanasia.

Fertilized one-cell embryos were injected with 200 pg pcDNA3 containing human HIF-2α cDNA (non-mutant) at a concentration of 100 ng/μl for the over-expression assay. Forty-eight hpf of embryos with pcDNA3 were distributed on a 6-well plate (30 embryos per well) (BD Biosciences, Franklin Lake, NJ, USA) to evaluate vessel formation in zebrafish embryos. Thalidomide (Sigma-Aldrich, St. Louis, MO, USA) was dissolved in DMSO. Thalidomide at 200, 400, and 800 μM was added to the zebrafish embryos. Control embryos were treated with equivalent amounts of DMSO. All embryos were incubated at 28.5 °C. Embryos were anaesthetised after treatment with 0.016% MS-222 (tricainemethanesulfonate, Sigma-Aldrich, St. Louis, MO). Embryos were observed and photographed using a Nikon SMZ 1500 fluorescence microscope (Nikon, Tokyo, Japan). The number of ectopic sprouts in the subintestinal vein (SIV) was counted. Quantitative image analyses were performed using morphometric analyses (NIS-Elements D3.1, Japan). A subset of images was adjusted for levels, brightness, contrast, hue and saturation using Adobe Photoshop 7.0 software (Adobe, San Jose, California) to optimally visualise the expression patterns.

### Statistical analysis

The data are expressed as the mean ± SEM and analysed using one-factor analysis of variance (ANOVA) followed by Tukey’s post hoc test. Statistical analyses of the data were performed using GraphPad Prism 5.0 (Graphpad Software, San Diego, CA, USA). Two-sided P-values < 0.05 were considered statistically significant.

## Additional Information

**How to cite this article**: Feng, N. *et al*. HIF-1α and HIF-2α induced angiogenesis in gastrointestinal vascular malformation and reversed by thalidomide. *Sci. Rep.* **6**, 27280; doi: 10.1038/srep27280 (2016).

## Supplementary Material

Supplementary Information
