# Supplementary material for: HIF-1α and HIF-2α induced angiogenesis in gastrointestinal vascular malformation and reversed by thalidomide
Source: Sci Rep. 2016 Jun 1;6:27280. doi: 10.1038/srep27280 (PMC4888746; doi:10.1038/srep27280)
Supplement: Supplementary Information [file srep27280-s1.pdf]

HIF-1α and HIF-2α induced angiogenesis in gastrointestinal vascular malformation and reversed by thalidomide

Nan Feng[1], Haiying Chen[1], Sengwang Fu[1], Zhaolian Bian[2], Xiaolu Lin[1], Li Yang[1], Yunjie Gao[1], Jingyuan Fang[1], Zhizheng Ge[1*]

[1] Key Laboratory of Gastroenterology & Hepatology, Ministry of Health, Division of Gastroenterology & Hepatology, Ren Ji Hospital, School of Medicine, Shanghai Jiao Tong University, Shanghai Institute of Digestive Diseases, 145 Middle Shandong Road, Shanghai 200001, China.

[2] Department of gastroenterology and hepatology, Nantong Institute of liver diease, Nantong Third people's Hospital, Nantong 223006, China

Nan Feng, Dr. Haiying Chen, Dr. Sengwang Fu contribute equally to this paper.

Correspondence to: Dr. Zhizheng Ge, Key Laboratory of Gastroenterology & Hepatology, Ministry of Health, Division of Gastroenterology & Hepatology, Ren Ji Hospital, School of Medicine, Shanghai Jiao Tong University, Shanghai Institute of Digestive Diseases, 145 Middle Shandong Road, Shanghai 200001, China. Tel: 86-21-58752345/Fax: 86-21-68383015, E-mail: zhizhengge@aliyun.com

Supplementary material 1. Primers for real-time PCR.

Gene *(Homo Sepiens)*	Primer sequence (5’ -> 3’)
GAPDH	Upstream: GCATTGCCCTCAACGACCAC
	Downstream: CCACCACCCTGTTGCTGTAG
HIF-1α	Upstream: TAGCCGAGGAAGAACTATGAAC
	Downstream: CACACTGAGGTTGGTTACTGTTG
HIF-2α	Upstream: AATCCGAGCAGTGGAGTCATT
	Downstream: ACCCTCTTGGCAGCAATAATAA
VEGF	Upstream: CCTCACACCATTGAAACCAC
	Downstream: AAGGGAAGGGAAGGACCAG
Notch1	Upstream: CGGAGTGTGTATGCCAAGAG
	Downstream: GGTTCTGGAGGGACCAAGA
DLL4	Upstream: CTGGAGGTGAGGTGAGTGCT
	Downstream: GGGTAGACGGACATTCTTGC
Ang2	Upstream: GAAAGAATGTGGCAGATTG
	Downstream: GGCAGGAGGAAAGTGTAG

Gene*(Danio rerio)*	Primer sequence (5’ -> 3’)
GAPDH	Upstream: GCATTGCCCTCAACGACCAC
	Downstream: CCACCACCCTGTTGCTGTAG
HIF-2α	Upstream: AGAGCGGCGTAAGGAGAAAT

	Downstream: GCTGATGCTGTGTGGAAGG
VEGF	Upstream: CCGTCCTGTGTGGTTCTCAT
	Downstream: GCTTTGACTTCTGCCTTTGG
Notch1a	Upstream: CATCTCAGCCGTGTCTCAAC
	Downstream: CATTCATCCACATCCACCTG
Notch1b	Upstream: GCAGATTCCCTCTCCGTGTA
	Downstream: CTCTCGCAGTGTTTCCCAGT
Notch2	Upstream: CTCCACCTTCTTGCCCTTATC
	Downstream: TCACTTCCATCCCAGTCACA
Notch3	Upstream: ATGGCACTTACACCACCACA
	Downstream: CCCTCCTCCACAGAAAGATG
DLL4	Upstream: AGTGGGCTGCTGTTTCTCTG
	Downstream: CGAGTCCTTTCTCCTGATGC
